# Cardiac Muscarinic Receptor Overexpression in Sudden Infant Death Syndrome

**DOI:** 10.1371/journal.pone.0009464

**Published:** 2010-03-01

**Authors:** Angelo Livolsi, Nathalie Niederhoffer, Nassim Dali-Youcef, Caroline Rambaud, Catherine Olexa, Walid Mokni, Jean-Pierre Gies, Pascal Bousquet

**Affiliations:** 1 Laboratoire de Neurobiologie et Pharmacologie Cardiovasculaire, Université de Strasbourg, Strasbourg, France; 2 Pôle Pédiatrique Médico-Chirurgical, Hôpitaux Universitaires de Strasbourg, Strasbourg, France; 3 Laboratoire de Biophotonique et Pharmacologie, CNRS UMR 7213, Université de Strasbourg, Illkirch, France; 4 Institut de Génétique et de Biologie Moléculaire et Cellulaire de Strasbourg (IGBMC), INSERM/CNRS/Université de Strasbourg, Illkirch, France; 5 Laboratoire de Biochimie Générale et Spécialisée, Hôpitaux Universitaires, Strasbourg, France; 6 Service d'Anatomie Pathologique et Médecine Légale, Hôpital Raymond Poincaré, Garches, France; 7 Centre d'Investigation Clinique, Hôpitaux Universitaires/INSERM, Strasbourg, France; University of Giessen Lung Center, Germany

## Abstract

**Background:**

Sudden infant death syndrome (SIDS) remains the leading cause of death among infants less than 1 year of age. Disturbed expression of some neurotransmitters and their receptors has been shown in the central nervous system of SIDS victims but no biological abnormality of the peripheral vago-cardiac system has been demonstrated to date. The present study aimed to seek vago-cardiac abnormalities in SIDS victims. The cardiac level of expression of muscarinic receptors, as well as acetylcholinesterase enzyme activity were investigated.

**Methodology/Principal Findings:**

Left ventricular samples and blood samples were obtained from autopsies of SIDS and children deceased from non cardiac causes. Binding experiments performed with [^3^H]NMS, a selective muscarinic ligand, in cardiac membrane preparations showed that the density of cardiac muscarinic receptors was increased as shown by a more than doubled B_max_ value in SIDS (n = 9 SIDS *versus* 8 controls). On average, the erythrocyte acetylcholinesterase enzyme activity was also significantly increased (n = 9 SIDS *versus* 11 controls).

**Conclusions:**

In the present study, it has been shown for the first time that cardiac muscarinic receptor overexpression is associated with SIDS. The increase of acetylcholinesterase enzyme activity appears as a possible regulatory mechanism.

## Introduction

Sudden infant death syndrome (SIDS) is poorly understood and unpredictable. By definition, it refers to death in an infant less than 1 year of age when rigorous postmortem analysis fails to reveal an apparent cause. It remains the leading cause of death among infants aged between 1 month and 1 year [1]–[2]. In USA, SIDS accounted for 8.9% of all infant deaths, i.e. 7313 cases within the period 1999–2001 [3]. In Germany, in 2004, about 13% of all infant deaths, i.e. 394 cases, occurred suddenly and unexpectedly [4]. A prospective Italian study including 34,442 newborns showed that 0.7 ‰ died from SIDS [5]. It has been shown that the expression of several neurotransmitters and their receptors may be disturbed in the central nervous system of SIDS victims [6]–[10]. Several lines of evidence support the hypothesis that vagal stimulation might result from a central serotonin receptor overexpression [11]–[12]. Nicotine exposure during pregnancy has been proposed as the most common risk factor for SIDS [10]–[11].

To date, no biologic abnormality of the peripheral vago-cardiac system has been demonstrated [13]. Looking for such biological abnormalities, we assessed the cardiac muscarinic receptor expression level and the erythrocyte acetylcholinesterase (AchE) activity in tissue samples from children who died of SIDS compared to infants deceased from non cardiac causes (control group).

## Materials and Methods

### Human Tissues

Altogether 18 SIDS and 19 control infants were included in this study. Left ventricular samples (50–100 mg) (9 SIDS, 8 controls) and blood samples (9 SIDS, 11 controls) were obtained from children who died when aged between 1 and 9 months of age. SIDS was diagnosed according to the American Academy of Pediatrics definition: infant death that remains unexplained after a thorough case investigation, including performance of a complete autopsy, examination of the death scene, and review of the clinical history [2]. Infants with cardiac diseases (clinical history of heart disease and/or cardiac tissue abnormalities) were not diagnosed as SIDS and were not included in the control group. In the control group, the causes of death were: asphyxia (*n* = 5), infection (*n* = 7), dehydration (*n* = 3), metabolic disease (*n* = 1), shaken baby syndrome (*n* = 2) and sub-dural hematoma (*n* = 1). Groups were matched for gender (controls: 7F/12M; SIDS: 7F/11M) and age (controls: 4.0±2.3 months; SIDS: 4.5±1.5 months). Autopsies were carried out in the Service d'Anatomie Pathologique et Médecine Légale, Hôpital Raymond Poincaré (Garches, France). All autopsies, cardiac tissue examinations and tissue collections were performed by the same pathologist (CR) betweeen 1997 and 2007. Procedures were conducted in accordance with the French law (DGS/DH/225/2B, 1986, modified in 1987) and the recommendations of the Haute Autorité de Santé on SIDS handling. Informed parental consent for post-mortem examination and tissue collection for research purposes was obtained in all cases. All samples were analysed following a blinded procedure.

### Radioligand Binding Experiments

Methods were adapted from Gies et al. [14]–[15]. All saturation binding experiments were carried out at 25°C in 0.5 mL Tris buffer (50 mM, pH 7.4) containing 10–15 µg of protein. Samples were incubated for 90 min. Incubation was stopped by rapid vacuum filtration over Whatman GF/C glass microfibre filters (VWR International SAS, Strasbourg, France) presoaked with polyethylenimine 0.3% in order to reduce non-specific binding to the filters. Filters were washed twice with 4 mL of ice-cold incubation buffer and transferred to 6 mL counting vials containing 3 mL of scintillation cocktail (Ready Protein+™, Beckman Coulter France SA, Roissy CDG, France). Radioactivity was then counted in a liquid scintillation spectrometer (LS 6000SC; Beckman Instruments) with an efficiency of 45%. Scatchard analysis of the saturation data (linear regression with Excel Software) was used to yield the maximal specific binding sites (B_max_; fmol mg^−1^ protein) and the affinity constant (Kd; nmol L^−1^). Protein content was measured according to the method of Bradford [16] using bovine γ-globulin as standard.

The density of total muscarinic receptors was assessed using the tritiated non-selective muscarinic receptor antagonist N-methylscopolamine ([^3^H]NMS; 81.0 Ci mmol^−1^) added in 12 concentrations ranging from 40 to 2000 pmol L^−1^. Non-specific binding was determined in the presence of 1 µmol L^−1^ atropine.

### AchE Enzyme Activity

AchE enzyme activity was measured in erythrocytes from venous total blood samples collected on heparin according to an enzymatic colorimetric assay as previously described [17].

### Statistics

All values are expressed as mean ± standard deviation (SD). Unpaired *t*-tests were performed using the Mann-Whitney U test. *P* values = 0.05 were considered to be statistically significant.

## Results

**Figure 1:**
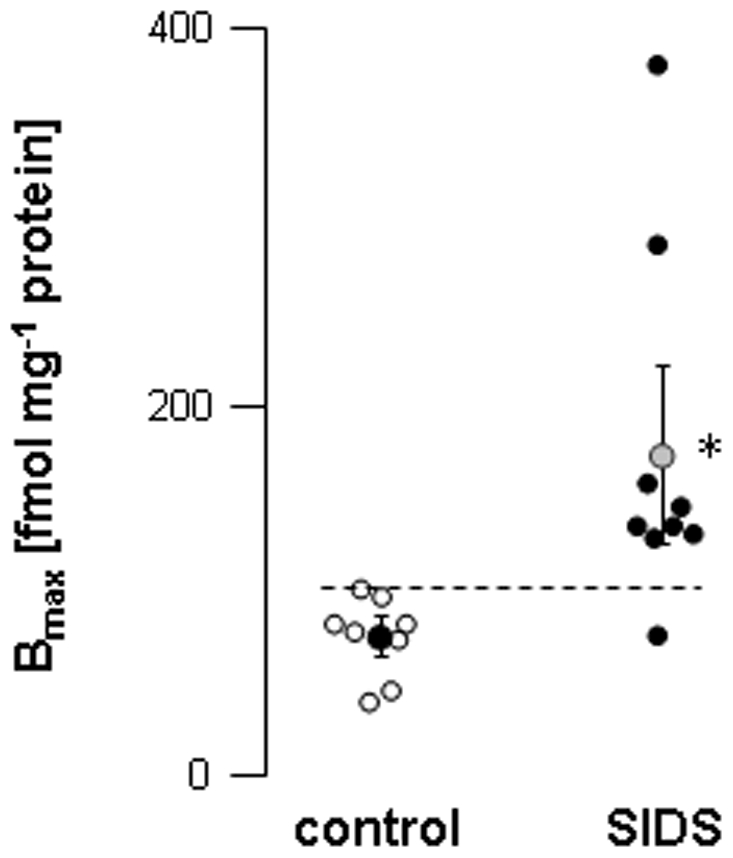
Total muscarinic receptors in hearts from control and SIDS children. Total muscarinic receptor densities (B_max_; fmol mg^−1^ protein) were obtained from Scatchard analysis of the saturation data using [^3^H]NMS as radioligand. Binding conditions were as described in Material and Methods. Each symbol represents one individual value; hatched bar represents the highest control value. *: *P* = 0.0137 *versus* control.

**Figure 2:**
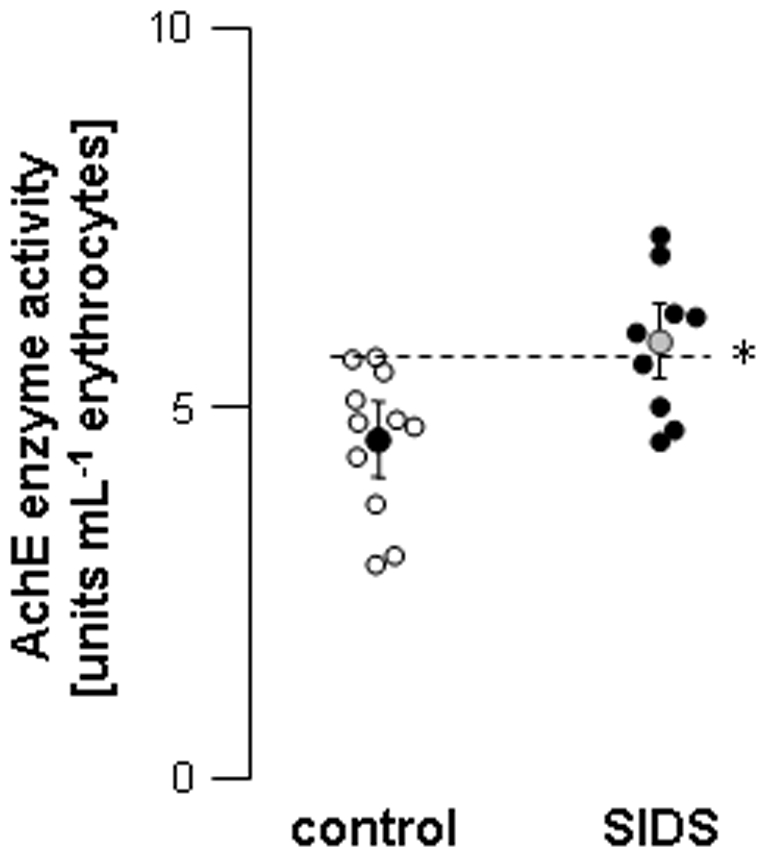
AchE enzyme activity in erythrocytes from control and SIDS children. AchE enzyme activity was assayed colorimetrically in erythrocyte hemolysates. Each symbol represents one individual value; hatched bar represents the highest control value. *: *P* = 0.0128 *versus* control.

**Figure 3:**
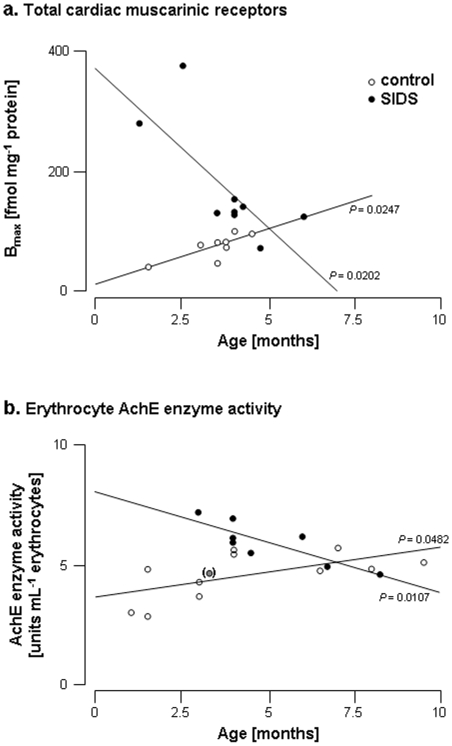
Total cardiac muscarinic receptors and erythrocyte AchE enzyme activity as a function of age in control and SIDS children. Total muscarinic receptor densities (B_max_; fmol mg^−1^ protein) were obtained from Scatchard analysis of the saturation data using [^3^H]NMS as radioligand. AchE enzyme activity was assayed colorimetrically in erythrocyte hemolysates. Each symbol represents one individual value. (**a**) Total cardiac muscarinic receptors and (**b**) Erythrocyte AchE enzyme activity in controls (open symbols) and SIDS (full symbols); the value in brackets indicates the only SIDS case which did not fit the AchE/age correlation.

In order to evaluate possible abnormalities of the cardiac vagal system involved in SIDS, the density of cardiac muscarinic receptors and the AchE activity levels were assessed in samples obtained from autopsied SIDS victims and compared to values measured in infants deceased from other causes (except from cardiac causes). In the latter (control group), cardiac muscarinic receptor density ranged from 46.1 to 100.5 fmol mg^−1^ protein and AchE activity ranged from 2.9 to 5.7 units mL^−1^ erythrocytes. In the control group, both parameters were homogenous whatever the cause of death. The total muscarinic receptor density in the heart of children who died from SIDS was more than double that found in samples from children who died from other causes (171.0±95.3 *versus* 74.5±21.5 fmol mg^−1^ protein; **Fig. 1**). Of note, values of muscarinic receptor density were above control in 8 out of 9 samples from SIDS (**Fig. 1**). The affinity constant Kd was similar in both groups (0.375±0.114 and 0.476±0.308 nmol L^−1^ in controls and SIDS, respectively). Average erythrocyte AchE activity was also significantly increased in SIDS victims (5.8±1 *versus* 4.6±1 units mL^−1^ erythrocytes; **Fig. 2**). In controls, the older the infants were, the higher cardiac muscarinic receptor density and AchE enzyme activity (**Fig. 3**). In contrast, the density of cardiac muscarinic receptors and AchE activity were negatively correlated with age in SIDS victims; only one case did not fit the AchE/age correlation (in brackets in **Fig. 3**).

## Discussion

In this study, we were seeking possible abnormalities in the intra-cardiac part of the vagal system associated with SIDS. To date, cholinergic abnormalities have been reported only in the central nervous system of SIDS victims: reduced muscarinic binding has been shown in the arcuate nucleus [7], a decrease in choline acetyltransferase immunoreactivity, a specific marker of the cholinergic neurons, has been observed in the brainstem [8] and alterations in nicotinic binding have been observed in brainstem nuclei of children exposed to maternal smoking during pregnancy [10]. In animals, a dysregulation of the autonomic balance toward vagal hyperactivity has been suggested as a possible consequence of nicotine exposure during pregnancy leading to SIDS [11]. Nevertheless, peripheral effectors of the vagal system are exclusively muscarinic receptors and non-neuronal cholinergic nicotinic receptors have not been detected so far in human hearts. Therefore, we focussed our investigations on muscarinic receptors only. We now show peripheral vago-cardiac abnormalities - namely overexpression of muscarinic receptors - in SIDS victims. Overexpression of cardiac muscarinic receptors and increased AchE activity in erythrocytes from SIDS were observed. All SIDS included in the present study were diagnosed according to the American Academy of Pediatrics definition, namely the clinically unexplainable death of an infant less than 1 year of age. Among 18 samples, only 4 came from infants older than 6 months, at which age social factors might contribute. However, all these individuals exhibited muscarinic disorders similar to the younger ones, suggesting that social factors, if any, could not account for the biological disorders observed in SIDS victims. The control group included infants deceased from several causes, among which asphyxia. It may be argued that hypoxia could modify the expression of the muscarinic receptors. However, cardiac muscarinic receptor density and erythrocyte AchE activity measured in these particular infants were not different from those of the whole control group. The triple risk model proposed by Filiano & Kinney [18] suggests that sudden death results from three simultaneously occurring factors: an underlying vulnerability, an exogenous stressor and being in a critical developmental period. Our results suggest that overexpression of muscarinic receptors in the heart may represent one aspect of vulnerability. Sleeping in the ventral position would then act as the fatal trigger [19]. Further studies will determine the developmental evolution of the muscarinic receptor abnormality described here. A peak of SIDS has been observed between 2 and 3 months of age [2]; it will be of particular interest to seek whether the vago-cardiac abnormalities that we are describing here are more pronounced during this period of life. Our data afford parts of an answer. Figure 3 demonstrates opposite evolution of cardiac muscarinic density and AchE activity with age in SIDS victims compared to controls, and suggests that the larger the alterations in vagal regulation, the earlier the age of death. Moreover, differences between controls and SIDS occur mostly before the age of 5–7 months, and it is well known that 90% of SIDS occurs within the 6 first postnatal months. At first glance, increases in AchE activity appear paradoxical. Pharmacologically, AchE upregulation may allow to oppose the increased muscarinic receptor density in order to maintain the sympatho-vagal balance. Thus, the primary cardiac abnormality is rather the overexpression of muscarinic receptors, whereas AchE upregulation is possibly a consequence of the latter. Consistently with this assumption, there was virtually no overlap between B_max_ individual values obtained from SIDS hearts compared with controls (1/9), while there was a quite large overlap for AchE activity (4/9). However, since cardiac and blood samples were obtained from different sets of SIDS, cardiac muscarinic receptor density could not be correlated with AchE activity. In SIDS victims, the compensatory mechanism could be not efficient enough to restore cardiac autonomic balance and prevent from death.

In conclusion, we showed an overexpression of muscarinic receptors in cardiac tissues from SIDS. It remains to be demonstrated whether or not increased number of binding sites reflects functional differences and whether or not such an abnormality associated with vagal excessive activity might contribute to SIDS. In addition, AchE activity level was above control level in SIDS. Cardiac muscarinic receptor overexpression may represent the biological vulnerability to SIDS evoked by Malloy [20].
